# Fat Attenuation Index of Renal Cell Carcinoma Reveals Biological Characteristics and Survival Outcome

**DOI:** 10.3389/fonc.2022.786981

**Published:** 2022-06-09

**Authors:** Hongkai Wang, Yu Wei, Xiaoxin Hu, Jian Pan, Junlong Wu, Beihe Wang, Hailiang Zhang, Guohai Shi, Xiaohang Liu, Jinou Zhao, Yao Zhu, Dingwei Ye

**Affiliations:** ^1^ Department of Urology, Fudan University Shanghai Cancer Center, Shanghai, China; ^2^ Department of Oncology, Shanghai Medical College, Fudan University, Shanghai, China; ^3^ Department of Pathology, Fudan University, Shanghai, China; ^4^ Department of Radiology, Fudan University Shanghai Cancer Center, Shanghai, China

**Keywords:** CT fat attenuation index, obesity paradox, renal cell carcinoma, immune micro-environment, adipocyte

## Abstract

**Purpose:**

The computed tomography fat attenuation index (FAI) is an ideal quantifiable imaging factor to identify the inflammation degree of peri-tumor adipose tissue. We aimed to verify whether FAI could reflect peri-tumor adipose inflammation, predict the survival outcome of renal cell carcinoma (RCC), and discover transcriptomic features of tumor tissues and adjacent adipocytes.

**Materials and Methods:**

Two clinical cohorts (Fudan University Shanghai Cancer Center [FUSCC] cohort [n=129] and TCGA cohort [n=218]) were used to explore the association between FAI and clinical outcome. A prospective cohort (n = 19) was used to discover the molecular phenotyping of peri-tumor adipose tissue and tumor tissue according to their FAI value. A clinical cohort (n = 32) in which patients received cyto-reductive surgery was used to reveal the dynamic change of FAI.

**Results:**

A high peri-tumor FAI was significantly associated with a worse outcome in both the FUSCC (HR = 2.28, p = 0.01) and the TCGA cohort (HR = 2.24, p <0.001). The analysis of the RNA expression of paired RCC tissue and peri-tumor fat tissue showed synchronized alterations in pathways such as cytokine–cytokine receptor interaction and complement and coagulation cascades. RCC tissues showed significant alterations in the neuroactive ligand–receptor interaction pathway. Immune deconvolution analysis showed enhanced infiltration of macrophages in high FAI tumor tissues with a lower angiogenesis level. We also observed synchronous dynamic changes in FAI and tumor size after targeted therapy.

**Conclusion:**

In summary, FAI could be used in RCC to reflect the biological characteristics and tumor immune micro-environment of both the tumor and the peri-tumor adipose. High peri-tumor FAI had the potential to predict a worse survival outcome in various cohorts. This study demonstrates that the crosstalk exists between a tumor and its micro-environment and could be reflected easily by imaging procedures, which could facilitate clinical decision making.

## Introduction

Obesity is associated with an increased incidence of renal cell carcinoma (RCC) (1). However, a high body mass index (BMI) is believed to be a protective factor for RCC prognosis (2). The association between obesity and RCC is quite complex. Adipose tissue may have played an important role in the “obesity paradox” since it has multiple physiologic and pathophysiologic functions. Albiges and colleagues tried to explain the paradox in cohorts of metastatic RCC, and found that the fatty acid synthase (FASN) pathway activation is associated with BMI and survival, which is linked to lipogenesis of the tumor (3). On the other hand, adipose tissues have paracrine functions by secreting adipokines such as adiponectin and leptin or by secreting inflammatory cytokines such as tumor necrosis factor-a (TNF-a), interleukin-6 (IL-6), interleukin-8 (IL-8), plasminogen activator inhibitor 1 (PAI1), etc., which could facilitate cancer growth (4). Recently, Sanchez and colleagues found that tumors of obese patients showed higher angiogenic scores and that inflammation in the peri-tumoral adipose tissue was increased in obese patients (5). These results showed that interactions exist between the tumor and peri-tumoral adipose tissues, and those interactions could be a good explanation for the obesity paradox.

It is crucial to understand how these interactions can be used in making clinical decisions and predicting outcomes. Hakimi et al. showed that different molecular subgroups of clear cell RCC, especially those with angiogenesis and macrophage infiltration, may be powerful predictors of outcome with tyrosine kinase inhibitor (TKI) efficacy (6). Clark and colleagues characterized the immune infiltration of clear cell RCC into four sub-types, discriminated by the presence or absence of cell types related to immune (CD8+ T cells, macrophages, dendritic cells) and stromal (fibroblast, endothelial) signatures. They announced that these sub-types could be leveraged to predict the therapeutic response, such as immunotherapy (7). Either immune infiltration or angiogenesis could release inflammatory mediators or oxidation products, which could directly modify the phenotype of peri-tumor adipocytes. Since multiple image-based scoring systems have been developed for RCC outcome prediction, namely, RENAL, PADUA, C-index, and Mayo Adhesive Probability Score, is it possible to simplify immune/angiogenesis variables to an easily obtained factor to predict the outcome? We noticed that Antonopoulos and colleagues had developed the computed tomography (CT) fat attenuation index (FAI), which has excellent sensitivity and specificity for detecting tissue inflammation in peri-vascular adipose tissue (8). The development of FAI is an ideal quantifiable factor to help us identify the exact inflammation degree of peri-tumor adipose tissue.

We verified whether FAI could be used in RCC to reflect peri-tumor adipose inflammation, discovered transcriptomic features of tumor tissues and adjacent adipocytes, and evaluated whether FAI could be a predictor of tumor biological characteristics and survival outcome in various cohorts.

## Methods

### Study Design, Inclusion Criteria, and Participants

**Figure 1:**
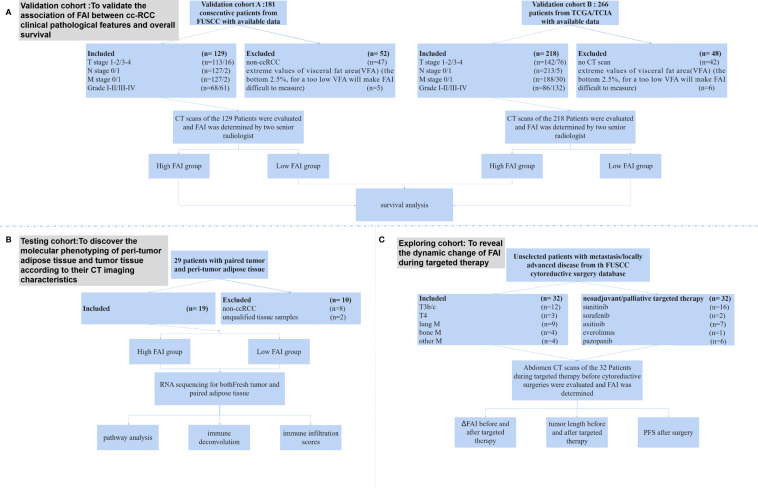
The study design, inclusion criteria, and participants. **(A)** The validation cohorts were composed by patients from the FUSCC database (FAI cohort A) and TCGA/TCIA database (FAI cohort **B**). We excluded patients who did not have CT scans and those who had extreme values of VFA. The final included patients were 129 from the FUSCC cohort and 218 from the TCGA/TCIA cohort. **(B)** The imaging genomics cohort prospectively included consecutive unselected patients from FUSCC who had nephrectomy or nephron sparing surgery. After excluding 8 patients who were not clear cell RCC and 2 patients with unqualified tissue samples, the final cohort included 19 patients. **(C)** The treatment response cohort included patients from a FUSCC cytoreductive surgery database. All patients had CT scans of the kidney tumor before and after two months of neoadjuvant/palliative targeted therapy, and then they undergone nephrectomies.

The study design, inclusion criteria, and participants are shown in **Figure 1**. In this study, we analyzed the data from four independent clinical cohorts. All cohorts included patients with clear cell RCC aged 18 years and older. There were no duplicated cases among those cohorts.

#### FAI Cohort A—FUSCC

For the FAI cohort A, all patients were from the Fudan University Shanghai Cancer Center (FUSCC, Shanghai, China) and had been histologically confirmed by surgery from November 2013 to November 2015. All patients had undergone contrast-enhanced CT of the abdomen before surgery. The clinical and pathological characteristics of the patients were collected from the database. All patients were confirmed as having clear cell RCC. The cohort used in this retrospective study was approved by the Ethics Committee of FUSCC.

#### FAI Cohort B—TCGA

For the FAI cohort B, all patients were from the TCGA and TCIA databases. Only patients with RNA sequencing data, CT scans before surgery, and pathology confirmed clear cell RCC were included. The survival data of those patients were achieved.

The two validation cohorts were designed to analyze and validate the association between FAI and clinical pathological features and the overall survival of those patients.

#### Imaging Genomics Cohort

For the imaging genomics cohort, we prospectively included consecutive unselected patients from FUSCC who underwent nephrectomy or nephron sparing surgery from July 2019 to September 2019. All patients had CT scans before surgery. Fresh tumor and adipose tissue collected from those patients were used for RNA sequencing. Those who were confirmed to be clear cell RCC were finally included in the study.

The imaging genomics cohort was to: 1) link molecular phenotyping of peri-tumor adipose tissue and tumor tissue with their CT imaging characteristics; 2) discover whether tumor-adipose interaction exists; and 3) observe how tumor immune micro-environment changes.

#### Treatment Response Cohort

For the treatment response cohort, we included patients from a FUSCC cyto-reductive surgery database. All patients had CT scans of the kidney tumor before and after two months of neo-adjuvant/palliative targeted therapy, and then they underwent nephrectomies. After that, targeted therapy was performed to further treat metastasis sites. The clinical and pathological characteristics of the patients were collected.

The treatment response cohort was to discover how FAI changes after targeted therapies.

### Imaging Studies Using CT

Fat attenuation index (FAI): Adipose tissue was defined as voxels with attenuation between −190 and −30 HU. Voxel attenuation histograms were plotted, and FAI was defined as the average attenuation of the adipose tissue volume of interest as previously described (8). FAI_PTAT_ was defined as the FAI of the first 5-mm-thick layer of peri-tumor adipose tissue (PTAT) (**Supplementary Figure 1**). All FAIs were determined by two senior radiologists who were blinded to patient characteristics and the mean count was adopted. The intraclass correlation coefficient between the two radiologists from the same CT scan was 0.927 (95% CI 0.909 to 0.937, P <0.001).

### Procedures

The RNA sequencing and data analysis of tumor tissue and peri-tumor fat from the imaging genomics cohort was described in **Supplementary Data 1**. GO (http://www.geneontology.org/) and KEGG (https://www.kegg.jp/) enrichment analyses of annotated different expressed genes were performed by Phyper (http://en.wikipedia.org/wiki/Hypergeometric_distribution) based on the Hypergeometric test. The protein–protein interaction (PPI) network was predicted using the STRING (http://string-db.org) (version 10.0) online database (9).

RNAseq data of the TCGA cohort, mainly of the tumor tissue, were downloaded from the National Institutes of Health Genomic Data Commons. The methods for RNA extraction and processing for the TCGA cohort have previously been published (10). For both the FUSCC and TCGA cohorts, ssGSEA for immune deconvolution analyses was used as previously described (11). The immune infiltration score, the fraction of immune cells (ImmuneScore), each individual immune cell type, angiogenesis score, and hypoxia score were calculated according to previous studies (5).

Multiplex immunofluorescence (mIF) was done to confirm the status of immune cell infiltration. The procedures are shown in **Supplementary Data 2**.

### Statistical Analysis

Continuous data are presented as the median (range), and binary data are presented as proportions. The association between FAI, clinical features, transcriptomic, and genomic differences was tested using Fisher’s exact tests, Pearson’s tests, and χ^2^ tests. The Kaplan–Meier method was used to determine the overall survival rate. Overall survival rates were compared using the log-rank test. Predictive parameters were assessed in the Cox proportional hazards model, and odds ratios with 95% confidence intervals were calculated. All other analyses were conducted using SPSS 20.0 software (IBM, Chicago, IL, USA). Two-tailed P-values were used, and a P <0.05 was considered to indicate statistical significance.

## Results

The FAI cohort A from FUSCC included 181 consecutive patients who had CT scans before surgery, adequate clinical–pathological data, and overall survival data (**Figure 1**). After excluding 47 non-ccRCC patients and 5 patients who had extreme values of visceral fat area (VFA) (the bottom 2.5%, for a VFA too low will make FAI difficult to measure), our final cohort comprised 129 patients. The FAI cohort B from TCGA/TCIA included 266 patients with CT/magnetic resonance imaging (MRI) images. We excluded 42 patients who only had MRI scans and 6 patients who had extreme values of VFA (the bottom 2.5%). Our final cohort of cohort B comprised 218 patients. In the imaging genomics cohort for RNA sequencing, we prospectively collected 29 pairs of tumor and peri-tumor adipose tissue from 29 consecutive patients. After excluding 8 patients who were not clear cell RCC and 2 patients with unqualified tissue samples, the final cohort included 19 patients (**Supplementary Table S1**). The treatment response cohort included 32 patients, of which 17 patients had metastasis and 15 patients had tumors that extended into the vena cava or adjacent organs (**Supplementary Table S2**).

### Higher FAI_PTAT_ Indicates Worse T Stage, M Stage, Tumor Grade, and Worse Overall Survival Both in the FAI Cohorts A and B

**Figure 2:**
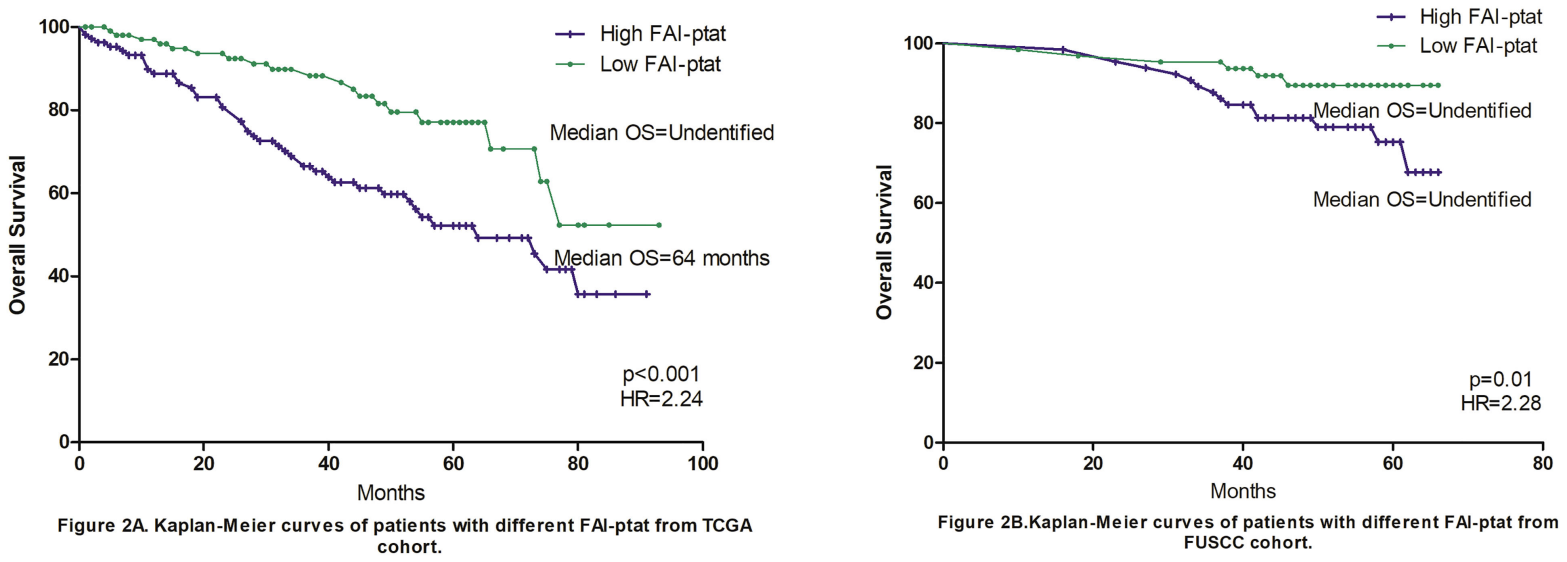
Kaplan–Meier curves of patients with different FAI_PTAT_. Fat attenuation index (FAI) was calculated and grouped according to the median FAT value of the FUSCC and TCGA cohorts. **(A)** Overall survival of the TCGA cohort, the 5-year median overall survival was 64 months for high FAI_PTAT_ group and not reached for low FAI_PTAT_ group (HR = 2.24, p <0.001). **(B)** Overall survival of the FUSCC cohort, the 5-year median overall survival was not reached for both groups in FUSCC cohort (HR = 2.28, p = 0.01). The 3-year survival rate was 88%/93% for high FAI_PTAT_ and low FAI_PTAT_ groups in FUSCC cohort (HR = 1.81) and 72%/93% in TCGA cohort (HR = 5.65).

Patients from the FAI cohort A (FUSCC) had fewer T3–4 stage patients, fewer M stage patients, a lower FAI_PTAT_ compared with the FAI cohort B (TCGA) cohort, as well as a lower rate of overweight and obese patients. FAI_PTAT_ tends to be lower in obese patients. However, no association with BMI was observed in either cohort (p = 0.29/p = 0.056). Data showed that FAI_PTAT_ were significantly associated with T stage (p = 0.028/p = 0.00), M stage (p = 0.01/p = 0.00), and tumor grade (p = 0.028/p = 0.00) in both the FUSCC and TCGA cohorts (**Table 1**). Necrosis status was available for the FUSCC cohort. Both necrosis (p = 0.01) and SSIGN score (p = 0.001) were significantly associated with FAI_PTAT_. We set the cutoff of FAI_PTAT_ to be −93 hu (median value, ranging from −33 to −113) for the FAI cohort A (FUSCC), and −79 hu (median value, ranging from −31 to −108) for the FAI cohort B (TCGA), and divided the cohorts into high FAI_PTAT_ and low FAI_PTAT_ groups. Multivariate analysis showed that FAI_PTAT_ (p = 0.007; p = 0.027) and M stage (p = 0.001; p = 0.006) were significantly associated with overall survival (**Table 2**) both in the TCGA and FUSCC cohorts. When Kaplan–Meier curves were made for the high FAI_PTAT_ and low FAI_PTAT_ groups, the 5-year median overall survival was not reached for both groups in the FUSCC cohort (HR = 2.28, p =0.01), while the median overall survival was 64 months for high FAI_PTAT_ group and not reached for the low FAI_PTAT_ group in the TCGA cohort (HR = 2.24, p <0.001) (**Figure 2**). We then calculated the 3-year survival rate. It was 88%/93% for high FAI_PTAT_ and low FAI_PTAT_ groups in the FUSCC cohort (HR =1.81) and 72%/93% for high FAI_PTAT_ and low FAI_PTAT_ groups in the TCGA cohort (HR = 5.65). Those results showed that FAI_PTAT_ may be an independent factor for overall survival.

**Table 1:** Clinical Pathological Characteristics of FAI.

Characteristics	TCGA (n = 218)			FUSCC (n = 129)		
	No. (N)	FAI_PTAT_	p value	No. (N)	FAI_PTAT_	p-value
Male	145	−76.9	0.78	87	−87.7	0.033
Female	73	−77.6		42	−94.3	
Age						
<64	142	−77.6	0.62	104	−90.4	0.44
>64	76	−76.3		25	−87.5	
T stage						
1–2	142	−82.5	<0.001	113	−91.1	0.028
3–4	76	−67.1		16	−81.4	
N stage						
0	213	−77.3	0.27	127	−90.1	0.27
1	5	−68.6		2	−77.1	
M stage						
0	188	−78.9	<0.001	127	−90.3	0.01
1	30	−66.3		2	−60.6	
Necrosis						
No	NA			94	−92.1	0.01
Yes				35	−83.7	
Grade						
I–II	86	−83.6	<0.001	68	−91.1	0.028
III–IV	132	−72.9		61	−81.4	
BMI						
normal	36	−74.4	0.056	60	−88.3	0.29
overweight	61	−74.1		64	−90.5	
obese	84	−80.3		5	−99.8	
Hypertension						
No	80	−77.9	0.56	88	−91.5	0.09
Yes	101	−76.4		41	−86.3	
Diabetes						
No	NA			112	−90.2	0.61
Yes				17	−87.9	
Smoking						
No	92	−78.1	0.41	77	−90.8	0.44
Yes	89	−75.9		52	−88.5	
SSIGN						
0–2	NA			81	−94.6	0.001
3–4				20	−83.9	
5–6				16	−82.9	
7–9				10	−78.1	
≥10				2	−70.1	

NA, not available.

**Table 2:** Univariate and Multivariate analysis for overall survival in patients with ccRCC.

Characteristics	Univariate analysis			Multivariate analysis		
	HR	95% CI	p-value	HR	95% CI	p-value
**TCGA cohort**						
Gender	1.46	0.88–2.42	0.14	0.93	0.52–1.69	0.82
Age	1.04	1.02–1.06	0.000	1.04	1.018–1.068	0.001
BMI	0.49	0.35–0.70	0.000	0.55	0.38–0.79	0.001
T stage	1.97	1.5–2.57	0.000	1.31	0.92–1.88	0.13
N stage	4.00	1.25–12.84	0.02	0.40	0.09–1.84	0.24
M stage	4.53	2.71–7.56	0.000	2.44	1.35–4.43	0.003
Pathological Grade	1.63	0.93–2.84	0.09	0.61	0.32–1.19	0.15
△FAI_PTAT_	2.33	1.36–4.01	0.002	1.28	0.61–2.72	0.51
FAI_PTAT_	2.81	1.59–4.96	0.000	2.14	1.15–3.99	0.017
**FUSCC cohort**						
Gender	1.57	0.66–3.73	0.31	2.49	0.97–6.16	0.056
Age, years	1.003	0.96–1.04	0.86	0.99	0.95–1.05	0.96
BMI	1.03	0.44–1.97	0.85	1.06	0.44–2.52	0.89
T stage	1.93	0.64–5.75	0.23	1.03	0.27–3.98	0.96
N stage	3.62	0.48–27.2	0.21	1.73	0.22–13.60	0.60
M stage	15.21	3.37–68.6	0.000	9.26	1.88–45.5	0.006
Pathological Grade	4.00	1.35–11.92	0.013	3.53	1.16–10.68	0.026
△FAI_PTAT_	2.42	0.94–6.23	0.068	1.18	0.34–4.11	0.79
FAI_PTAT_	3.31	1.21–9.05	0.019	3.14	1.11–8.88	0.031

HR, Hazard Ratio; CI, Confidence Interval; FAI, Fat Attenuation Index; BMI, Body Mass Index.

### Higher FAI_PTAT_ Indicates Pathway Alterations in the Tumor Tissue and the Peri-Tumor Adipose Tissue

#### A) Neuroactive Ligand–Receptor Interaction Was Altered in the Tumor Tissue Both in the Imaging Genomics Cohort and FAI Cohort B (TCGA)

**Figure 3:**
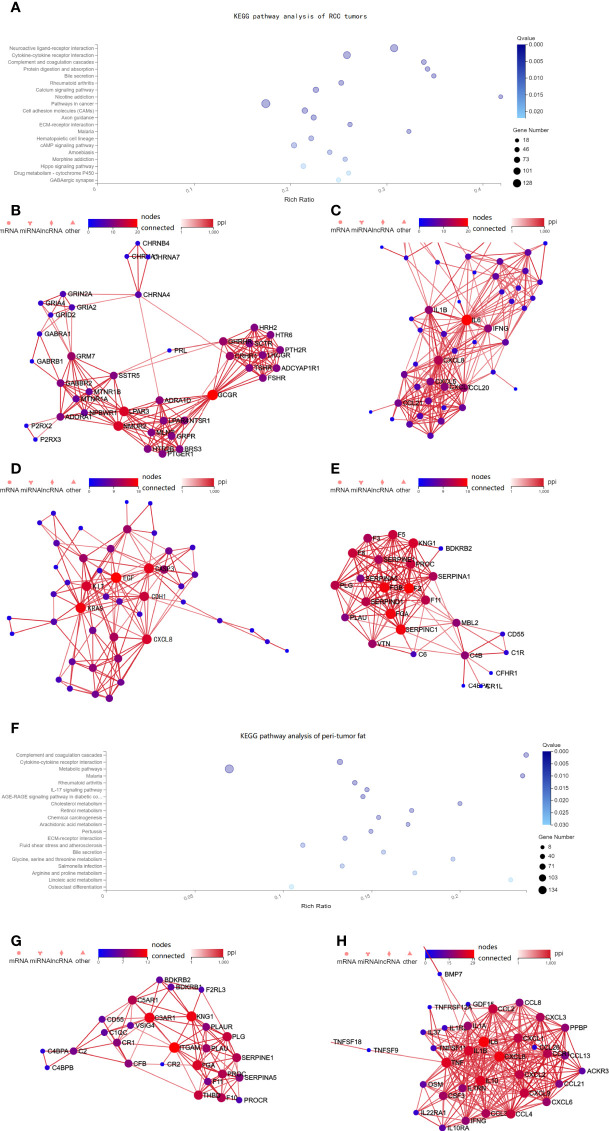
Pathway alterations in the tumor tissue from the imaging genomics cohort. **(A)** KEGG enrichment analysis of annotated different expressed gene was performed in the imaging genomics cohort. Pathway analysis showed that the neuroactive ligand–receptor interaction, cytokine–cytokine receptor interaction, complement and coagulation cascades were altered in high FAI_PTAT_ patients compared with low FAI_PTAT_ patients. **(B–E)** The PPI networks were drawn using Cytoscape and key genes from those pathways such as GCGR, LPAR3, NMUR2, EGF, KIT, KRAS, CXCL8, IL-6, GCGR, FGA, and FGB were observed to be altered. **(F)** Pathway analysis showed that the complement and coagulation cascades, cytokine–cytokine receptor interaction, and multiple metabolic pathways were altered in high FAI_PTAT_ patients compared with low FAI_PTAT_ patients. **(G–H)** The PPI networks were drawn using Cytoscape, key genes such as ITGAM, FGA, and KNG1 were observed for the complement and coagulation cascades, while alterations of IL-6, TNF, CXCL8, IL-19, CXCL2, IL-1B, etc. were observed for the cytokine pathway.

We then further explored the potential mechanism by assessing transcriptomic differences in the tumor tissue using the imaging genomics cohort and FAI cohort B (TCGA). In the imaging genomics cohort, KEGG pathway analysis showed that tumors of the high FAI_PTAT_ group had significant alterations in pathways such as neuroactive ligand–receptor interaction, cytokine–cytokine receptor interaction, complement and coagulation cascades, and pathways of cancer (**Figure 3A**). The PPI networks were drawn using Cytoscape and key genes from those pathways such as glucagon receptor (GCGR), Lysophosphatidic Acid Receptor 3 (LPAR3), Neuromedin U Receptor 2 (NMUR2), Epidermal Growth Factor (EGF), Proto-oncogene c-KIT (KIT), Kirsten rat sarcoma virus (KRAS), C-X-C Motif Chemokine Ligand 8 (CXCL8), IL-6, GCGR, Fibrinogen Alpha Chain (FGA), and Fibrinogen Beta Chain (FGB) were observed (**Figures 3B–E**). The pathway results were confirmed by the FAI cohort B from the TCGA database, which also presented neuroactive ligand–receptor interaction as the most significantly altered pathway (**Supplementary Figure 2**).

#### B) Complement and Coagulation Cascades, Cytokine–Cytokine Receptor Interaction and Multiple Metabolic Pathways Were Altered in the Peri-Tumor Adipose Tissue in the Imaging Genomics Cohort

Transcriptomic differences in the peri-tumor adipose tissues were available using the imaging genomics cohort. Peri-tumor adipose tissue with higher FAI values showed significant alteration in pathways such as complement and coagulation cascades, cytokine–cytokine receptor interaction and multiple metabolic pathways (cholesterol metabolism, retinol metabolism, glycine, serine and threonine metabolism, arginine and proline metabolism, linoleic acid metabolism, etc.) (**Figure 3F**). Strong alterations of Integrin Subunit Alpha M (ITGAM), FGA, and kininogen 1 (KNG1) were observed for the complement and coagulation cascades (**Figure 3G**), while alterations of IL-6, Tumor Necrosis Factor (TNF), C-X-C Motif Chemokine Ligand 8 (CXCL8), interleukin 19 (IL-19), C-X-C Motif Chemokine Ligand 2 (CXCL2), interleukin 1 beta (IL-1B), etc. were observed for the cytokine pathway (**Figure 3H**).

Thus, FAI_PTAT_ could not only represent the characteristics of the tumor but also reveal the alterations of the peri-tumor adipose tissue. In the imaging genomics cohort, it was surprising that the cytokine–cytokine receptor interaction and the complement and coagulation cascades were both altered in the tumor and adipose tissues.

### Higher FAI_PTAT_ Indicates Diverse Immune Micro-Environment in the Tumor Tissue and the Peri-Tumor Adipose Tissue

#### A) Antigen Presenting and Angiogenesis may be Enhanced in Tumors of High FAI Patients Both in the Imaging Genomics Cohort and the TCGA Validation Cohort

**Figure 4:**
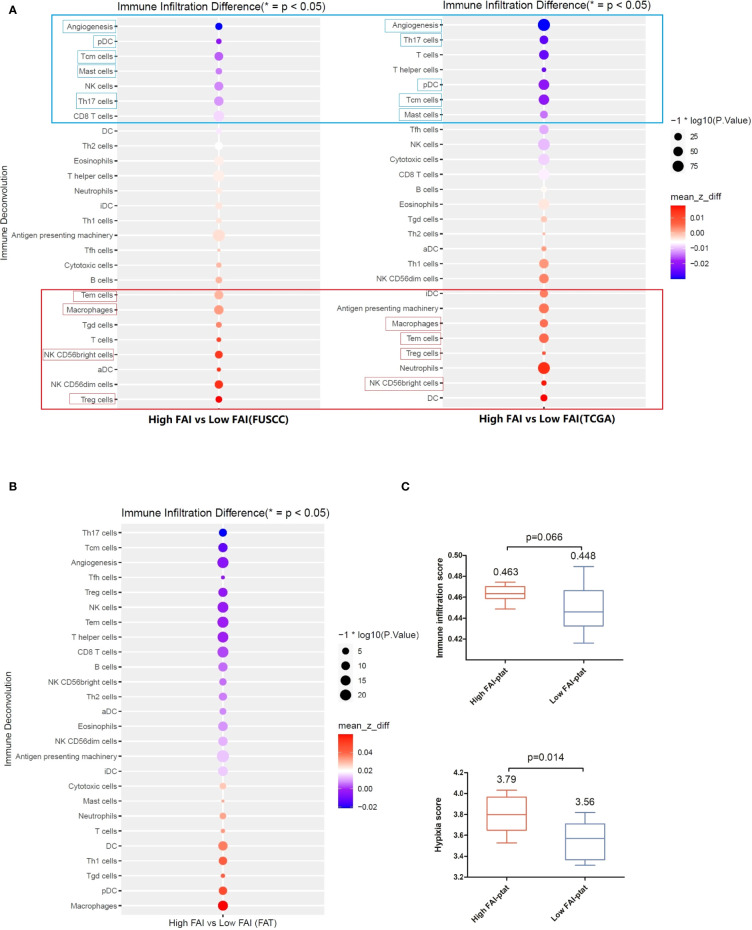
The immune microenvironment of tumor tissue of the imaging genomics cohort (FUSCC) and the FAI cohort B (TCGA). Immune deconvolution showed good consistency between the both cohort. **(A)** Tumors of high FAI_PTAT_ patients showed enhanced infiltration of Treg, NK, CD56bright cells, Tem cells, and macrophages, and a reduced infiltration of pDC, Tcm cells, Th17 cells, and mast cells, as well as a lower angiogenesis level compared with low FAI_PTAT_ patients. **(B)** Immune deconvolution showed an enhanced infiltration of macrophages, pDC cells, Th1 cells, and DC cells, and a reduced infiltration of Th17 cells, Tcm cells, Treg cells, CD8+ T cells, and NK cells, as well as a lower angiogenesis level in peri-tumor fat. **(C)** Hypoxia score and immune infiltration score were enhanced in fat of high FAI_PTAT_ patients.

We then analyzed the immune micro-environment of tumor tissue from the imaging genomics cohort (FUSCC) and the FAI cohort B (TCGA) by using immune deconvolution. We found good consistency between both the cohorts, tumors of high FAI_PTAT_ patients showed enhanced infiltration of regulatory T cells (Treg), natural killer (NK), CD56 bright cells, effector memory T cells (Tem), and macrophages. Additionally, dendritic cells (DCs) were seen enhanced in the TCGA group, and B cells were seen enhanced in the FUSCC group. Reduced infiltration of central memory T cells (Tcms), T helper 17 cell (Th17) cells, and mast cells (**Figure 4A**) and a lower angiogenesis level were observed. We believe that antigen presentation was enhanced as macrophage, DC cells, and B cells were the main functional cells of antigen presentation. These results were then confirmed by multiplex immunofluorescence (mIF) in ten patients, that high FAI_PTAT_ tumors may have more infiltrated macrophages and T cells (**Supplementary Figure 3**).

#### B) High Hypoxia Score, Immune Infiltration Score and Macrophage Infiltration Scores Were Observed in Fat Tissues of High FAI Patients in the Imaging Genomics Cohort

Fat tissues from the imaging genomics cohort were available. Immune deconvolution showed enhanced infiltration of macrophages, pDC, Th1, DC, and reduced infiltration of Th17, Tcm, Treg, CD8+ T, and NK (**Figure 4B**), as well as a lower angiogenesis level. Hypoxia score and immune infiltration score were enhanced in the fat of high FAI_PTAT_ patients compared with low FAI_PTAT_ patients (**Figure 4C**).

### FAI_PTAT_ Decreases After Effective Targeted Therapy

**Figure 5:**
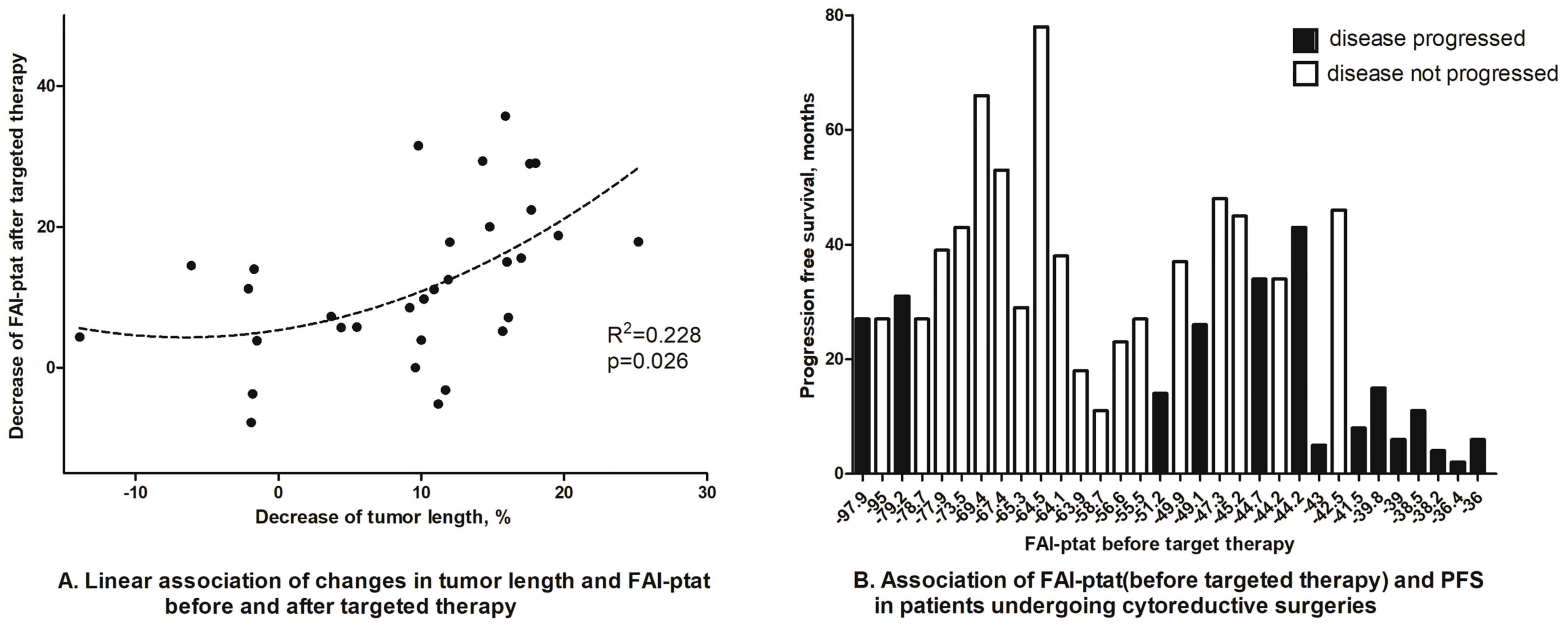
Dynamic changes of FAI_PTAT_ after targeted therapy. **(A)** Scattergram showed that FAI_PTAT_ changes synchronously with tumor size after targeted therapy. **(B)** Waterfall plot showed that patients with high FAI_PTAT_ had short PFS during targeted therapy.

Finally, using the treatment response cohort, we tried to observe the dynamic changes of FAI_PTAT_ after neo-adjuvant/palliative targeted therapy in patients who subsequently underwent nephrectomies and whether FAI_PTAT_ could be altered by RCC treatment. Scattergram showed that there was an association between a decrease in FAI_PTAT_ after targeted therapy and a decrease in tumor size (**Figure 5A**). Waterfall plot showed that patients with high FAI_PTAT_ before targeted therapy will progress sooner; however, due to the limited number of participants, no significant statistics could be made (**Figure 5B**).

## Discussion

In this study, we introduced a fat attenuation index imaging metric, which could quantify the degree of peri-tumor inflammation in renal cell carcinoma. We used four distinct cohorts to verify the outcome prediction ability of FAI in RCC patients and tried to discover the transcriptomic features of tumor tissues and adjacent adipocytes in high/low FAI groups. Our observations suggest that a high FAI_PTAT_ (which indicates high inflammation status of peri-tumor fat) was significantly associated with a worse outcome in both the FUSCC and TCGA cohorts, and it also indicated short PFS in patients who undertook cyto-reductive surgeries after targeted therapies. Molecular analysis of RNA expression of paired RCC tissue and peri-tumor fat tissue showed synchronized alterations in pathways such as cytokine–cytokine receptor interaction and complement and coagulation cascades. RCC tissues from the TCGA and FUSCC cohorts both showed significant alterations in the neuroactive ligand–receptor interaction pathway. Additionally, immunodeconvolution analysis showed enhanced infiltration of macrophages in high FAI_PTAT_ tumor tissues with lower angiogenesis levels, consistent with previous studies indicating that Angio^low^Macrophage^high^ patients had worse outcomes (6). We also observed synchronous dynamic changes in FAI_PTAT_ and tumor size after targeted therapy, indicating that the tumor micro-environment would change after effective anti-cancer treatments. Whether the dynamic changes of FAI_PTAT_ are the cause or results of primary tumor response should be further explored, but the strength of the link between FAI_PTAT_ and RCC progression has improved.

CT imaging measurement of peri-tumor fat has been widely used in the assessment of adherent peri-nephric fat (APF), or so-called “sticky fat”, which would make partial nephrectomy difficult. The mayo adhesive probability (MAP) included posterior peri-nephric fat thickness and stranding and showed accuracy in predicting APF (12). Soon after, Thiel et al. reported that MAP may represent visceral obesity/inflammation and was associated with RCC prognosis (13). However, the introduction of stranding was quite subjective, and on the other hand, few had studied the relationship between stranding and inflammation as well as its molecular biological relationship with kidney tumors. FAI was initially introduced by Antonopoulos and colleagues to quantify vascular inflammation in peri-vascular adipose tissue by using CT imaging (8). They believe that high FAI indicates immature adipocytes with smaller sizes and less lipid accumulation, which is caused by inflammatory signals released by the coronary artery (14). Oikonomou et al. further indicated that high peri-vascular FAI values (cutoff ≥−70·1 HU) were an indicator of increased cardiac mortality (15). Since FAI has been proven to be an objective, reproducible, and quantifiable factor, we believe it could demonstrate the peri-tumor fat stranding and inflammation degree. We tried to investigate how FAI could reflect the phenotypic character of peri-tumor adipose tissue and whether it could somehow reflect the tumor characteristics.

Kidney tumors with high FAI_PTAT_ showed alterations in multiple pathways. The neuroactive ligand–receptor interaction pathway was the most significantly altered pathway of tumors in both the FUSCC and TCGA databases. After constructing the PPI network using Cytoscape, we observed that GCGR, LPAR3, and NMUR2 were the most significant modules in the pathway. GCGR is a receptor for glucagon and plays a central role in the regulation of blood glucose levels, glucose homeostasis, amino acid metabolism, and lipid metabolism (16). Further investigation is needed to determine whether glucagon metabolism plays a certain role in RCC development. Other key genes such as EGF, KIT, KRAS, CXCL8, and IL-6 were also shown to be altered, all of which indicated worse outcomes and could affect the effectiveness of TKIs (17–19). Research has indicated that up-regulation of the pro-inflammatory cytokines IL-6, TNF-a, and IFN-γ secreted by cancer tissues could prevent the differentiation of pre-adipocytes and cause-altered phenotypes. Those so-called cancer-associated adipocytes could then contribute to promoting tumor aggressiveness by over-expression of pro-inflammatory cytokines [interleukin (IL)-6, IL-1β] and vice versa (20). According to our results, we observed an activated cytokine pathway both in the tumor tissue and the paired fat tissue, promoting strong interaction between the tumor and peri-tumor fat tissue. Thus, peri-tumor fat morphology represented by FAI_PTAT_ could present the tumor characteristics such that inflammation enhancement both in the tumor itself and the peri-tumor environment could be observed in highly aggressive kidney tumors.

We then tried to evaluate the immune infiltration of kidney tumors and peri-tumor fat in high FAI_PTAT_ patients. We observed high consistency of immune infiltration of tumor tissue in the FUSCC and TCGA cohorts. For high FAI_PTAT_ patients in both cohorts, higher scores of Treg, NK, CD56bright cells, Tem cells, macrophages, and antigen-presenting machinery were observed in tumor tissue; and a lower number of pDC, Tcm cells, Th17 cells, mast cells, and a lower angiogenesis status were observed. Considering that high FAI_PTAT_ patients had short PFS after targeted therapy in the treatment response cohort, these results were supported by the research done by Hakimi et al., which showed that Angio^low^Macrophage^high^ represented worse survival and poor TKI sufficiency (6). Immune deconvolution of peri-tumor fat tissue also showed higher scores of macrophages, pDC cells, Th1 cells, and DC cells in high FAI_PTAT_ patients, as well as lower scores of Th17 cells, Tcm cells, Treg cells, CD8+ T cells, NK cells, and angiogenesis. Macrophages are often the most abundant cell type in the tumor micro-environment, among which tumor associated macrophages (TAM) support angiogenesis, tissue remodeling, and immune suppression. It was reported that RCC tumors with high-infiltration TAMs were significantly associated with poor prognosis (18). One of the possible theories was that TAMs may accumulate in regions of hypoxia, and the initial hypoxic response may cause M2 polarization, which has pro-tumorigenic functions (21). Another significantly altered immune cell were Treg cells, which also suppress anti-tumor immune responses. There is solid evidence that infiltration of Treg cells is often associated with poor prognosis and poor immunotherapy effectiveness (22). However, due to its high expression of CTLA-4, it is presumed an anti-CTLA-4 antibody may reduce Treg cells and make immunotherapy more effective. Thus, understanding the relationship between FAI_PTAT_ and the tumor micro-environment may help decision-making. However, further evaluations are needed.

This study had certain limitations. 1) The cohort size was quite small for both clinical analysis and RNA sequencing. However, the biological characteristics were validated and had good consistency between different cohorts. 2) We only included clear-cell RCC; further exploration should be made for non-clear RCC. 3) The immune infiltration status of immune cells was mainly calculated by immune deconvolution and immune infiltration scores, IHC was done for 10 patients. However, they were not validated by flow cytometry. 4) We also had concerns about whether the outcome distinguished by FAI_PTAT_ was due to peri-nephric fat invasion. Landman and colleagues indicated that peri-nephric soft-tissue stranding was a significant factor for predicting peri-nephric fat invasion, especially in tumors 4 cm or less (23). However, the data was so controversial that Bradley et al. reported that the presence of peri-nephric stranding and tumor necrosis were not reliable signs for pT stage >T3a (24). On the other hand, the survival differences between T3a and T2 tumors were not so significant (25). We would rather believe that the survival differences between high FAI_PTAT_ and low FAI_PTAT_ patients were due to tumor transcriptomic characteristics or the micro-environments represented by peri-tumor fat.

In summary, FAI could be used in RCC to reflect the inflammation status of both the tumor and the peri-tumor adipose. Additionally, FAI has the potential to predict tumor biological characteristics and survival outcomes in various cohorts. This study demonstrates that the crosstalk exists between a tumor and its micro-environment and could be reflected easily by imaging procedures and then facilitates clinical decision making.

## Data Availability Statement

The datasets presented in this study can be found in online repositories. The names of the repository/repositories and accession number(s) can be found in the article/**Supplementary Material**.

## Ethics Statement

The studies involving human participants were reviewed and approved by the Human Ethics Committee of Fudan University Shanghai Cancer Center. The patients/participants provided their written informed consent to participate in this study. Written informed consent was obtained from the individual(s) for the publication of any potentially identifiable images or data included in this article.

## Author Contributions

YZ and DWY contributed to conception and design of the study. HW and YZ organized the database. YW, XH, HZ, GS, HL, and JOZ performed the statistical analysis. JP, BHW and JLW participated in laboratory experiments. HW wrote the first draft of the manuscript. YW and YZ wrote sections of the manuscript. All authors listed have made a substantial, direct, and intellectual contribution to the work and approved it for publication.

## Funding

The manuscript is supported by the National Natural Science Foundation of China Projects 81972375 and 81802528.

## Conflict of Interest

The authors declare that the research was conducted in the absence of any commercial or financial relationships that could be construed as a potential conflict of interest.

## Publisher’s Note

All claims expressed in this article are solely those of the authors and do not necessarily represent those of their affiliated organizations, or those of the publisher, the editors and the reviewers. Any product that may be evaluated in this article, or claim that may be made by its manufacturer, is not guaranteed or endorsed by the publisher.

## References

[1] Renehan AG Tyson M Egger M Heller RF Zwahlen M. Body-Mass Index and Incidence of Cancer: A Systematic Review and Meta-Analysis of Prospective Observational Studies. Lancet (2008) 371(9612):569–78. doi: 10.1016/S0140-6736(08)60269-X 18280327

[2] Choi Y Park B Jeong BC Seo SI Jeon SS Choi HY. Body Mass Index and Survival in Patients With Renal Cell Carcinoma: A Clinical-Based Cohort and Meta-Analysis. Int J Cancer (2013) 132(3):625–34. doi: 10.1002/ijc.27639 22610826

[3] Albiges L Hakimi AA Xie W McKay RR Simantov R Lin X. Body Mass Index and Metastatic Renal Cell Carcinoma: Clinical and Biological Correlations. J Clin Oncol (2016) 34(30):3655–63. doi: 10.1200/JCO.2016.66.7311 PMC5065111 27601543

[4] Khandekar MJ Cohen P Spiegelman BM. Molecular Mechanisms of Cancer Development in Obesity. Nat Rev Cancer (2011) 11(12):886–95. doi: 10.1038/nrc3174 22113164

[5] Sanchez A Furberg H Kuo F Vuong L Ged Y Patil S. Transcriptomic Signatures Related to the Obesity Paradox in Patients With Clear Cell Renal Cell Carcinoma: A Cohort Study. Lancet Oncol (2019) 21(2):283–93. doi: 10.1016/S1470-2045(19)30797-1 PMC7082892 31870811

[6] Hakimi AA Voss MH Kuo F Sanchez A Liu M Nixon BG. Transcriptomic Profiling of the Tumor Microenvironment Reveals Distinct Subgroups of Clear Cell Renal Cell Cancer: Data From a Randomized Phase Iii Trial. Cancer Discovery (2019) 9(4):510–25. doi: 10.1158/2159-8290.CD-18-0957 PMC6697163 30622105

[7] Clark DJ Dhanasekaran SM Petralia F Pan J Song X Hu Y. Integrated Proteogenomic Characterization of Clear Cell Renal Cell Carcinoma. Cell (2019) 179(4):964–83.e31. doi: 10.1016/j.cell.2019.10.007 31675502 PMC7331093

[8] Antonopoulos AS Sanna F Sabharwal N Thomas S Oikonomou EK Herdman L. Detecting Human Coronary Inflammation by Imaging Perivascular Fat. Sci Trans Med (2017) 9(398):eaal2658. doi: 10.1126/scitranslmed.aal2658 28701474

[9] Szklarczyk D Franceschini A Wyder S Forslund K Heller D Huerta-Cepas J. String V10: Protein-Protein Interaction Networks, Integrated Over the Tree of Life. Nucleic Acids Res (2015) 43:D447–52. doi: 10.1093/nar/gku1003 PMC4383874 25352553

[10] Cancer Genome Atlas Research N. Comprehensive Molecular Characterization of Clear Cell Renal Cell Carcinoma. Nature (2013) 499(7456):43–9. doi: 10.1038/nature12222 PMC3771322 23792563

[11] Barbie DA Tamayo P Boehm JS Kim SY Moody SE Dunn IF. Systematic Rna Interference Reveals That Oncogenic Kras-Driven Cancers Require Tbk1. Nature (2009) 462(7269):108–12. doi: 10.1038/nature08460 PMC2783335 19847166

[12] Davidiuk AJ Parker AS Thomas CS Leibovich BC Castle EP Heckman MG. Mayo Adhesive Probability Score: An Accurate Image-Based Scoring System to Predict Adherent Perinephric Fat in Partial Nephrectomy. Eur Urol (2014) 66(6):1165–71. doi: 10.1016/j.eururo.2014.08.054 25192968

[13] Thiel DD Davidiuk AJ Meschia C Serie D Custer K Petrou SP. Mayo Adhesive Probability Score Is Associated With Localized Renal Cell Carcinoma Progression-Free Survival. Urology (2016) 89:54–60. doi: 10.1016/j.urology.2015.10.034 26723183

[14] Grant RW Stephens JM. Fat in Flames: Influence of Cytokines and Pattern Recognition Receptors on Adipocyte Lipolysis. Am J Physiol Endocrinol Metab (2015) 309(3):E205–13. doi: 10.1152/ajpendo.00053.2015 26058863

[15] Oikonomou EK Marwan M Desai MY Mancio J Alashi A Hutt Centeno E. Non-Invasive Detection of Coronary Inflammation Using Computed Tomography and Prediction of Residual Cardiovascular Risk (the Crisp Ct Study): A *Post-Hoc* Analysis of Prospective Outcome Data. Lancet (2018) 392(10151):929–39. doi: 10.1016/S0140-6736(18)31114-0 PMC6137540 30170852

[16] Janah L Kjeldsen S Galsgaard KD Winther-Sorensen M Stojanovska E Pedersen J. Glucagon Receptor Signaling and Glucagon Resistance. Int J Mol Sci (2019) 20(13):3314. doi: 10.3390/ijms20133314 PMC6651628 31284506

[17] Lai Y Zhao Z Zeng T Liang X Chen D Duan X. Crosstalk Between Vegfr and Other Receptor Tyrosine Kinases for Tki Therapy of Metastatic Renal Cell Carcinoma. Cancer Cell Int (2018) 18:31. doi: 10.1186/s12935-018-0530-2 29527128 PMC5838927

[18] de Vivar Chevez AR Finke J Bukowski R. The Role of Inflammation in Kidney Cancer. Adv Exp Med Biol (2014) 816:197–234. doi: 10.1007/978-3-0348-0837-8_9 24818725

[19] Fitzgerald JP Nayak B Shanmugasundaram K Friedrichs W Sudarshan S Eid AA. Nox4 Mediates Renal Cell Carcinoma Cell Invasion Through Hypoxia-Induced Interleukin 6- and 8- Production. PLoS One (2012) 7(1):e30712. doi: 10.1371/journal.pone.0030712 22303451 PMC3267761

[20] Dirat B Bochet L Dabek M Daviaud D Dauvillier S Majed B. Cancer-Associated Adipocytes Exhibit an Activated Phenotype and Contribute to Breast Cancer Invasion. Cancer Res (2011) 71(7):2455–65. doi: 10.1158/0008-5472.CAN-10-3323 21459803

[21] Quail DF Joyce JA. Microenvironmental Regulation of Tumor Progression and Metastasis. Nat Med (2013) 19(11):1423–37. doi: 10.1038/nm.3394 PMC3954707 24202395

[22] Tanaka A Sakaguchi S. Regulatory T Cells in Cancer Immunotherapy. Cell Res (2017) 27(1):109–18. doi: 10.1038/cr.2016.151 PMC5223231 27995907

[23] Landman J Park JY Zhao C Baker M Hofmann M Helmy M. Preoperative Computed Tomography Assessment for Perinephric Fat Invasion: Comparison With Pathological Staging. J Comput Assist Tomogr (2017) 41(5):702–7. doi: 10.1097/RCT.0000000000000588 28296683

[24] Bradley AJ MacDonald L Whiteside S Johnson RJ Ramani VA. Accuracy of Preoperative Ct T Staging of Renal Cell Carcinoma: Which Features Predict Advanced Stage? Clin Radiol (2015) 70(8):822–9. doi: 10.1016/j.crad.2015.03.013 25953656

[25] Siemer S Lehmann J Loch A Becker F Stein U Schneider G. Current Tnm Classification of Renal Cell Carcinoma Evaluated: Revising Stage T3a. J Urol (2005) 173(1):33–7. doi: 10.1097/01.ju.0000146719.43269.e8 15592020

