# Supplementary material for: Fat Attenuation Index of Renal Cell Carcinoma Reveals Biological Characteristics and Survival Outcome
Source: Front Oncol. 2022 Jun 9;12:786981. doi: 10.3389/fonc.2022.786981 (PMC9218210; doi:10.3389/fonc.2022.786981)

**Supplement**

**Fat attenuation index of renal cell carcinoma reveals biological characteristics and survival outcome**

Hongkai Wang^a,b, #^, Yu Wei^a,b, #^, Xiaoxin Hu^b,c, #^ , Hailiang Zhang^a,b^, Guohai Shi^a,b^,

Xiaohang Liu^a,b^, Jinou Zhao.^a,b^, Yao Zhu^a,b,*^, Dingwei Ye^a,b,*^

#: These authors contributed equally to this work.

* Corresponding authors

^a^ Department of Urology, Shanghai Cancer Center, Fudan University, Shanghai, China

^b^ Department of Oncology, Shanghai Medical College, Fudan University, Shanghai, China

c Department of Radiology, Fudan University Shanghai Cancer Center, Shanghai, China.

**Corresponding author and contact information:**

Ding-Wei Ye, M.D; Yao Zhu, M.D.

Department of Urology

Fudan University Shanghai Cancer Center

No. 270 Dong’an Road, Shanghai, 200032

People’s Republic of China

Email Address: [dwyeli@163.com](mailto:dwyeli@163.com); yaozhu09@fudan.edu.cn

Tel: 86-21-64175590-2807

Fax: 86-21-64434556

**Supplementary data 1.**

Total RNA of tumor and peri-nephric fat tissues (exactly near the tumor) from the imaging genomics cohort was extracted using Trizol (Invitrogen, Carlsbad, CA, USA) according to manual instruction, and then qualified and quantified using a Nano Drop and Agilent 2100 bioanalyzer (Thermo Fisher Scientific, MA, USA). Then first-strand cDNA was generated using SuperScriptTM Preamplification System for First Strand cDNA Synthesis according to manufacture instructions (Gibco, the USA). cDNA was then amplified by polymerase chain reaction (PCR) with the following cycling condition: initial denaturation in 98℃ for 45s, 12 PCR cycles (denaturation in 98℃ for 10s, annealing in 65℃ for 15s, extension in 72℃ for 3min), final extension in 72℃ for 5min,to obtain adequate cDNA. Afterwards, A-Tailing Mix and RNA Index Adapters were added by incubating to end repair. The cDNA fragments obtained from previous step were amplified by PCR, and products were purified by Ampure XP Beads, then dissolved in EB solution. The product was validated on the Agilent Technologies 2100 bioanalyzer for quality control. The double stranded PCR products from previous step were heated denatured and circularized by the splint oligo sequence to get the final library. The single strand circle DNA was formatted as the final library. The final library was amplified with phi29 to make DNA nanoball (DNB) witch had more than 300 copies of one molecular, DNBs were loaded into the patterned nanoarray and single end 50 bases reads were generated on BGIseq500 platform (BGI-Shenzhen, China).

The sequencing data was filtered with SOAPnuke(v 1.5.2) , afterwards clean reads were obtained and stored in FASTQ format. The clean reads were mapped to the reference genome using HISAT2 (v 2.0.4). Bowtie2 (v 2.2.5) was applied to align the clean reads to the reference coding gene set, then expression level of gene was calculated by RSEM (v 1.2.12). Differential expression analysis was performed using the PossionDis with False Dicovery Rate (FDR)≤0.001 and ｜Log2Ratio｜≥1.

**Supplementary data 2.**

Multiplex immunofluorescence staining was conducted using the Akoya OPAL Polaris 7-Color Automation IHC kit (NEL871001KT). FFPE tissue slides were first deparaffinized in a BOND RX system (Leica Biosystems) and then incubated sequentially with primary antibodies targeting CD163 (Abcam, ab182422, 1:500), CD68 (Abcam, ab213363, 1:1000), PD-1 (CST, D4W2J, 86163S, 1:200), PD-L1 (CST, E1L3N, 13684S, 1:400)， CD3 (Dako, A0452), CD4 (Abcam, ab133616, 1:100), CD8 (Abcam, ab178089, 1:100), CD56 (Abcam, ab75813, 1:100), CD20 (Dako, L26, IR604), FOXP3 (Abcam, ab20034, 1:100) and pan-CK ( Abcam, ab7753, 1:100) or S100 (Abcam, ab52642, 1:200) (Akoya Biosciences). This was followed by incubation with secondary antibodies and corresponding reactive Opal fluorophores. Nuclei acids were stained with DAPI. Tissue slides that were bound with primary and secondary antibodies but not fluorophores were included as negative controls to assess autofluorescence. Multiplex stained slides were scanned using a Vectra Polaris Quantitative Pathology Imaging System (Akoya Biosciences) at 20 nm wavelength intervals from 440 nm to 780 nm with a fixed exposure time and an absolute magnification of ×200. All scans for each slide were then superimposed to obtain a single image. Multilayer images were imported to inForm v.2.4.8 (Akoya Biosciences) for quantitative image analysis. Tumor parenchyma and stroma were differentiated by Pan-CK staining. The quantities of various cell populations were expressed as the number of stained cells per square millimeter and as the percentage of positively stained cells in all nucleated cells.

**Table S1.** Patient characteristics of the testing cohort.

**Table S2.** Patient characteristics of the exploring cohort.

A separate Excel file is created to represent information in these table, named **Supplementary** **Table**.

**Figure S1.** FAIPTAT was defined as the FAI of the first 5-mm- thick layer of peri-tumor adipose tissue (PTAT).


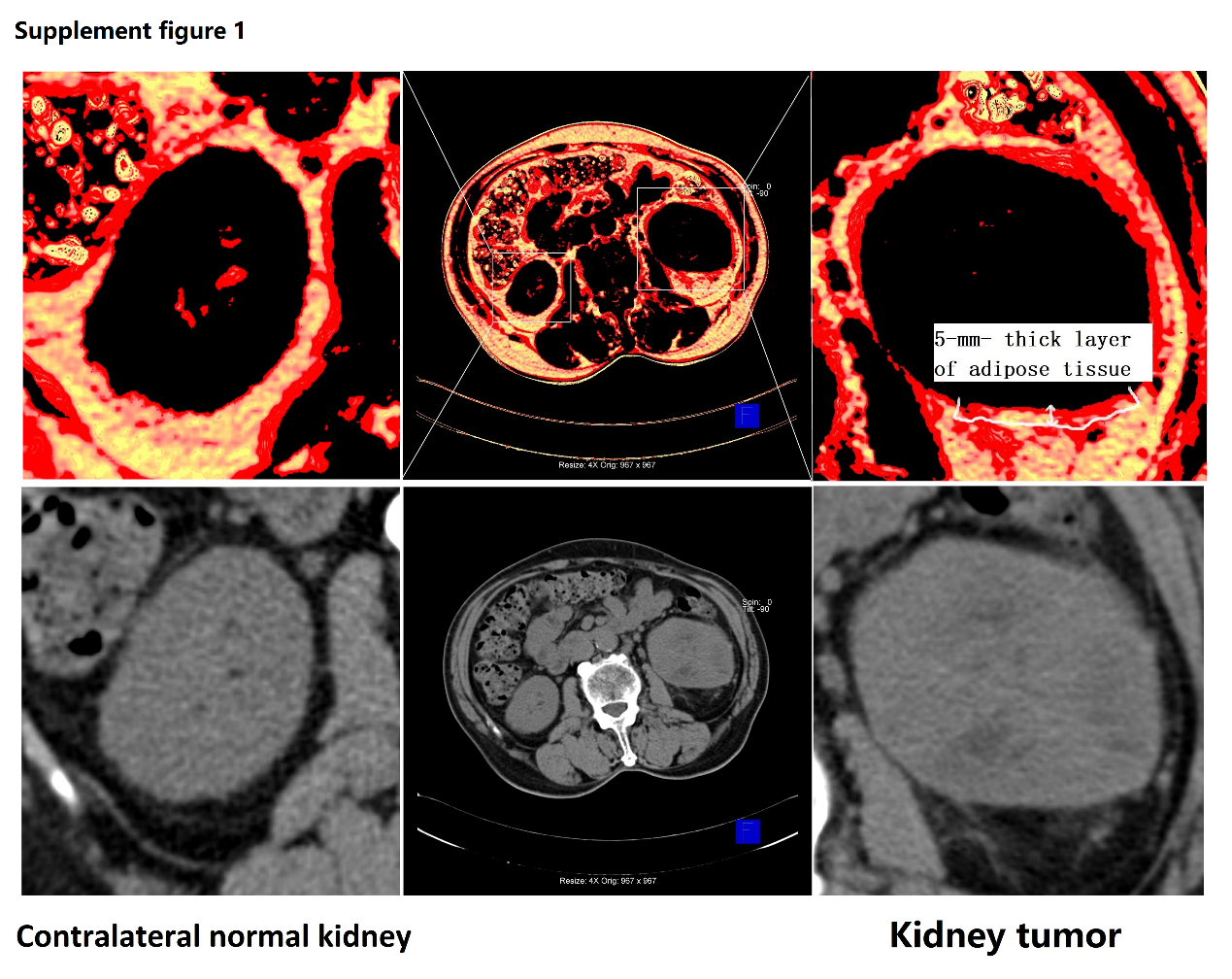


**Figure S2.** A) KEGG enrichment analysis of annotated different expressed gene was performed in the TCGA cohort. Results showed that the Neuroactive ligand-receptor interaction as the most significantly altered pathway, consistent with the FUSCC results.


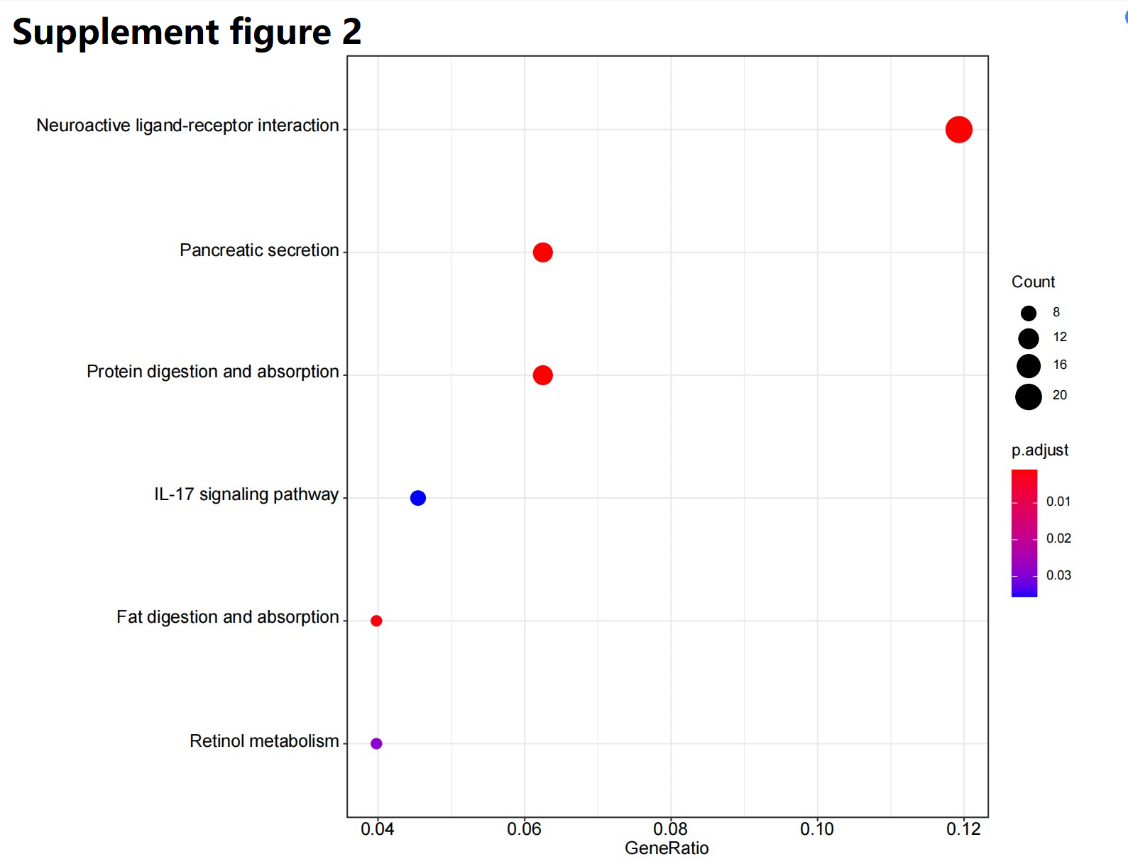

Supplement: Supplementary file 1 [file DataSheet_1.docx]
