# Supplementary figures and images for: Fat Attenuation Index of Renal Cell Carcinoma Reveals Biological Characteristics and Survival Outcome
Source: Front Oncol. 2022 Jun 9;12:786981. doi: 10.3389/fonc.2022.786981 (PMC9218210; doi:10.3389/fonc.2022.786981)

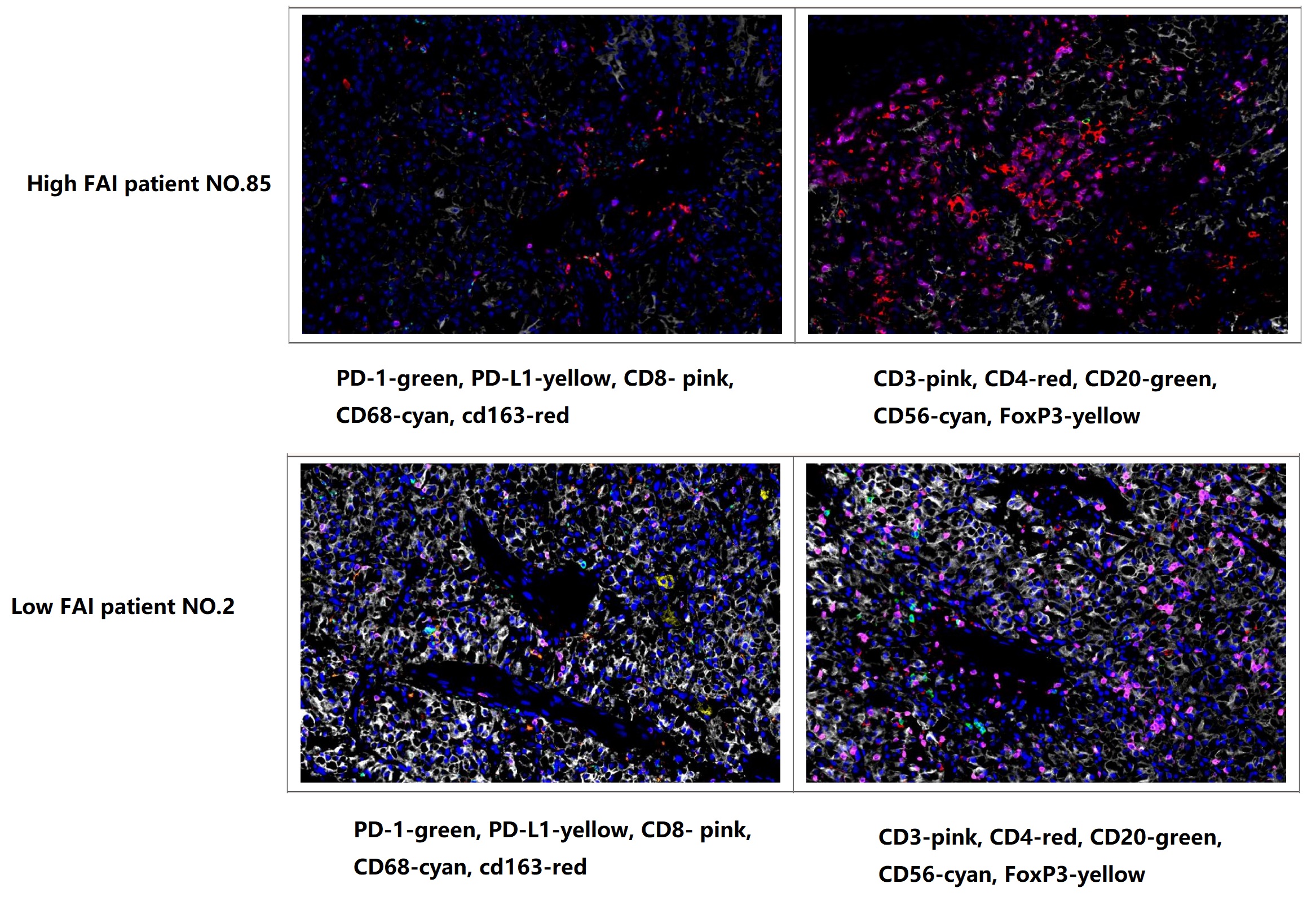

Supplement: Supplementary file 3 [file Image_1.jpeg]
